# Deep-learning segmentation of fascicles from microCT of the human vagus nerve

**DOI:** 10.3389/fnins.2023.1169187

**Published:** 2023-05-10

**Authors:** Ozge N. Buyukcelik, Maryse Lapierre-Landry, Chaitanya Kolluru, Aniruddha R. Upadhye, Daniel P. Marshall, Nicole A. Pelot, Kip A. Ludwig, Kenneth J. Gustafson, David L. Wilson, Michael W. Jenkins, Andrew J. Shoffstall

**Affiliations:** ^1^Department of Biomedical Engineering, Case Western Reserve University, Cleveland, OH, United States; ^2^Advanced Platform Technologies Center, Louis Stokes Cleveland VA Medical Center, Cleveland, OH, United States; ^3^Department of Biomedical Engineering, Duke University, Durham, NC, United States; ^4^Department of Biomedical Engineering, University of Wisconsin Madison, Madison, WI, United States; ^5^Department of Neurological Surgery, University of Wisconsin Madison, Madison, WI, United States; ^6^Wisconsin Institute for Translational Neuroengineering, Madison, WI, United States; ^7^Functional Electrical Stimulation Center, Louis Stokes Cleveland VA Medical Center, Cleveland, OH, United States; ^8^Department of Pediatrics, School of Medicine, Case Western Reserve University, Cleveland, OH, United States

**Keywords:** vagus nerve, microCT, image processing, segmentation, neural network, autonomic nervous system, deep-learning segmentation of vagus nerve

## Abstract

**Introduction:**

MicroCT of the three-dimensional fascicular organization of the human vagus nerve provides essential data to inform basic anatomy as well as the development and optimization of neuromodulation therapies. To process the images into usable formats for subsequent analysis and computational modeling, the fascicles must be segmented. Prior segmentations were completed manually due to the complex nature of the images, including variable contrast between tissue types and staining artifacts.

**Methods:**

Here, we developed a U-Net convolutional neural network (CNN) to automate segmentation of fascicles in microCT of human vagus nerve.

**Results:**

The U-Net segmentation of ~500 images spanning one cervical vagus nerve was completed in 24 s, versus ~40 h for manual segmentation, i.e., nearly four orders of magnitude faster. The automated segmentations had a Dice coefficient of 0.87, a measure of pixel-wise accuracy, thus suggesting a rapid and accurate segmentation. While Dice coefficients are a commonly used metric to assess segmentation performance, we also adapted a metric to assess fascicle-wise detection accuracy, which showed that our network accurately detects the majority of fascicles, but may under-detect smaller fascicles.

**Discussion:**

This network and the associated performance metrics set a benchmark, using a standard U-Net CNN, for the application of deep-learning algorithms to segment fascicles from microCT images. The process may be further optimized by refining tissue staining methods, modifying network architecture, and expanding the ground-truth training data. The resulting three-dimensional segmentations of the human vagus nerve will provide unprecedented accuracy to define nerve morphology in computational models for the analysis and design of neuromodulation therapies.

## 1. Introduction

The vagus nerve is a major autonomic pathway that carries signals to and from the brainstem and the visceral organs (Neuhuber and Berthoud, 2021). As a result of the role of the vagus nerve in regulating parasympathetic functions, vagus nerve stimulation (VNS) holds tremendous potential for treating numerous medical conditions. VNS is FDA-approved to treat epilepsy, depression, obesity, and after-effects of stroke (FDA, 1997, 2005, 2015, 2021; LivaNova, 2017). However, despite the heterogeneity of physiological functions of the vagus nerve, VNS generally involves placing a cuff electrode that wraps around the mid-cervical nerve trunk, which then results in activation of fibers causing both therapeutic benefit and potentially therapy-limiting side effects (Settell et al., 2020b; Blanz et al., 2023; Jayaprakash et al., 2023). To inform improved neuromodulation approaches that reduce side effects, the functional organization of the vagus nerve must be better understood (Settell et al., 2020a; Osanlouy et al., 2021; NIH, 2022). There is additional inherent value in understanding the vagus nerve’s functional pathways for the purpose of basic physiology and elucidation of its micro-anatomical features (Upadhye et al., 2022).

Anatomically-realistic computational models enable predictive simulations of nerve fiber activation in response to VNS, studies of mechanisms of action, and rational development of novel electrode designs and stimulation parameters for selective and effective stimulation (Musselman et al., 2021). The modeled nerve morphology is defined using the segmentation of a 2D histological cross section that is extruded to define the 3D finite element model (Musselman et al., 2021). However, a recent study used microCT to quantify the 3D fascicular morphology of the human vagus nerve and found that fascicles merged or split every 0.56 mm on average, with high intra- and inter-sample variability (Upadhye et al., 2022); thus, over the 8 mm center-to-center span of the clinical VNS cuff, these microCT data indicate that the 2D extrusion model does not reflect the true anatomy.

The fascicles and epineurium must be segmented from the raw microCT images to quantify anatomical metrics and to serve as inputs to computational models. Manual segmentation is highly time-consuming and is subject to user-to-user variability, which can be somewhat alleviated by semi-automated methods, such as Otsu’s thresholding or region growing, followed by manual correction (Gonzalez and Woods, 2018). Thus far, in our hands, common semi-automatic segmentation methods have under-performed in this application due to the non-uniformity of contrast in the fascicles and imaging artifacts.

Conversely, convolutional neural networks (CNNs) have been widely used in image classification and image detection applications due to their ability to recognize features without manual feature extraction or additional image processing steps (Gu et al., 2018). CNN-based image segmentation can distinguish features from both background and artifacts that would cause other automatic segmentations to fail (Sarma and Gupta, 2021; Kumar, 2023). Network architectures such as U-Net (Ronneberger et al., 2015), FPN-Feature Pyramid Network (Lin et al., 2017) and Mask R-CNN (He et al., 2017) have been designed to perform image segmentation. Further, execution of the CNNs on graphics processing units (GPUs) has greatly reduced training time (Alzubaidi et al., 2021). In recent years, countless applications of CNN-based image segmentation for medical images have been reported in the literature, from segmentation of CT (Li et al., 2022), MRI (Zhao and Zhao, 2021), ultrasound (Liu et al., 2019), optical coherence tomography (Viedma et al., 2022) or histology images (Basu et al., 2023), and from structures as varied as blood vessels (Chen et al., 2020, 2021), cells (Kumar et al., 2017; Stringer et al., 2021), and nerves(Balsiger et al., 2018; Wang et al., 2019; Horng et al., 2020; Tovbis et al., 2020; Kim et al., 2022; Jayaprakash et al., 2023).

In this work, we trained a U-Net CNN to achieve efficient and reproducible segmentation of fascicles from microCT images of the human cervical vagus nerve. We quantified its performance compared to ground-truth manual segmentation using multiple metrics, including Dice coefficient—which is a measure of pixel-wise detection accuracy—and adapted methods to create a new measure of fascicle-detection accuracy (Caicedo et al., 2019). A trained researcher requires several hours to segment microCT images of a single human cervical vagus nerve, whereas our trained CNN required seconds. The U-Net architecture is one of the most established and widely used CNN-based segmentation algorithms, and therefore may serve as a benchmark for future refinements.

## 2. Materials and methods

### 2.1. Sample acquisition and preparation

Human cervical vagus nerves were collected and prepared using methods from a previous study (Upadhye et al., 2022). Briefly, nerves were dissected and harvested from five de-identified cadavers (three left and five right sides) donated to the Case School of Medicine Anatomy Department (Cleveland, OH). Additional demographic information was not collected. A non-human subject determination was obtained from the Case Western Reserve University Institutional Review Board (IRB). The dissection was performed by a trained neuro-anatomy teaching assistant and vagus nerve sections from the jugular foramen to the clavicle were extracted. A total of five nerves from three subjects were chosen for this study based on the staining quality, and demographic information such as age and gender were not recorded.

Specimens were stored in 10% neutral buffered formalin (Fisher Scientific) for several days prior to subsequent processing. The authors would like to note that in subsequent studies, nerves have been collected from fixed cadavers and stored directly in 1X phosphate buffered saline (1X-PBS) with 0.01% sodium azide to avoid over-fixation.

A specimen labeling scheme is presented in the figures throughout this manuscript. Specimens were labeled based on subject number (1, 2, 3…) followed by the letter R (right) or L (left) to indicate the side of the body. Samples were stained with 1% (v/v) osmium tetroxide solution (Polysciences, IL, United States) as previously described (Upadhye et al., 2022). The nerves were embedded in paraffin and placed inside a plastic mold with grooves marked with radio-opaque paint every 5 mm to facilitate navigation during imaging.

### 2.2. Imaging

Nerves were scanned using a Quantum GX2 micro-computed tomography (micro-CT) scanner (Perkin Elmer, Waltham, MA, United States), with an excitation voltage of 90 kV, a current of 80 μA, a scan time of 14 min, and a scanning field of view of 36 mm in diameter and 20 mm in length. Three to four overlapping scans (minimum 20% overlap in the dimension along the nerve) were performed to capture at least 5 cm of vagus nerve length, centered approximately at the mid-cervical region where neuromodulation cuff electrodes are typically placed.

Image reconstruction was performed at 10 μm voxel resolution using the Rigaku software (Perkin Elmer, Waltham, MA, United States). The software limits reconstructions to a 512 × 512 × 512 voxel cube at a time, thus the reconstruction field of view was a sub-volume of size 5.12 × 5.12 × 5.12 mm. The resulting data were exported as 16-bit TIFF images. The sub-volumes were then down-sampled 10x in the dimension along the nerve length (i.e., to 512 × 512 × 51 pixels) by copying every 10th image into a new directory in an effort to reduce memory and processing requirements for subsequent steps. Subsequently, these volumes were stitched using ImageJ (FIJI, Version 2.1.0/1.53c). Slices in the final stitched dataset had voxel dimensions of 10 × 10 × 100 μm.

### 2.3. Ground truth creation

Images were imported into Simpleware^™^ ScanIP S-2021.06 (Synopsys, Mountain View, CA, United States). The nerve fascicles and the epineurium were segmented by a trained user using built-in region painting tools. Fascicles were segmented manually on each image (501 images per 5 cm nerve sample) due to the high amount of variability in brightness, contrast, and position from one slice to the other along the length of the nerve. In comparison, the shape of the epineurium was more uniform and thus it was manually segmented every 10th slice and a built-in interpolation algorithm was used to segment the epineurium in the remaining slices. Layers containing the segmentation masks were exported as binary images. At this stage, images where the whole epineurium was not contained within the image field-of-view were removed from the dataset (7 images from nerve 2 l, 57 images from nerve 6 l).

### 2.4. Image pre-processing and data augmentation

**Figure 1:**
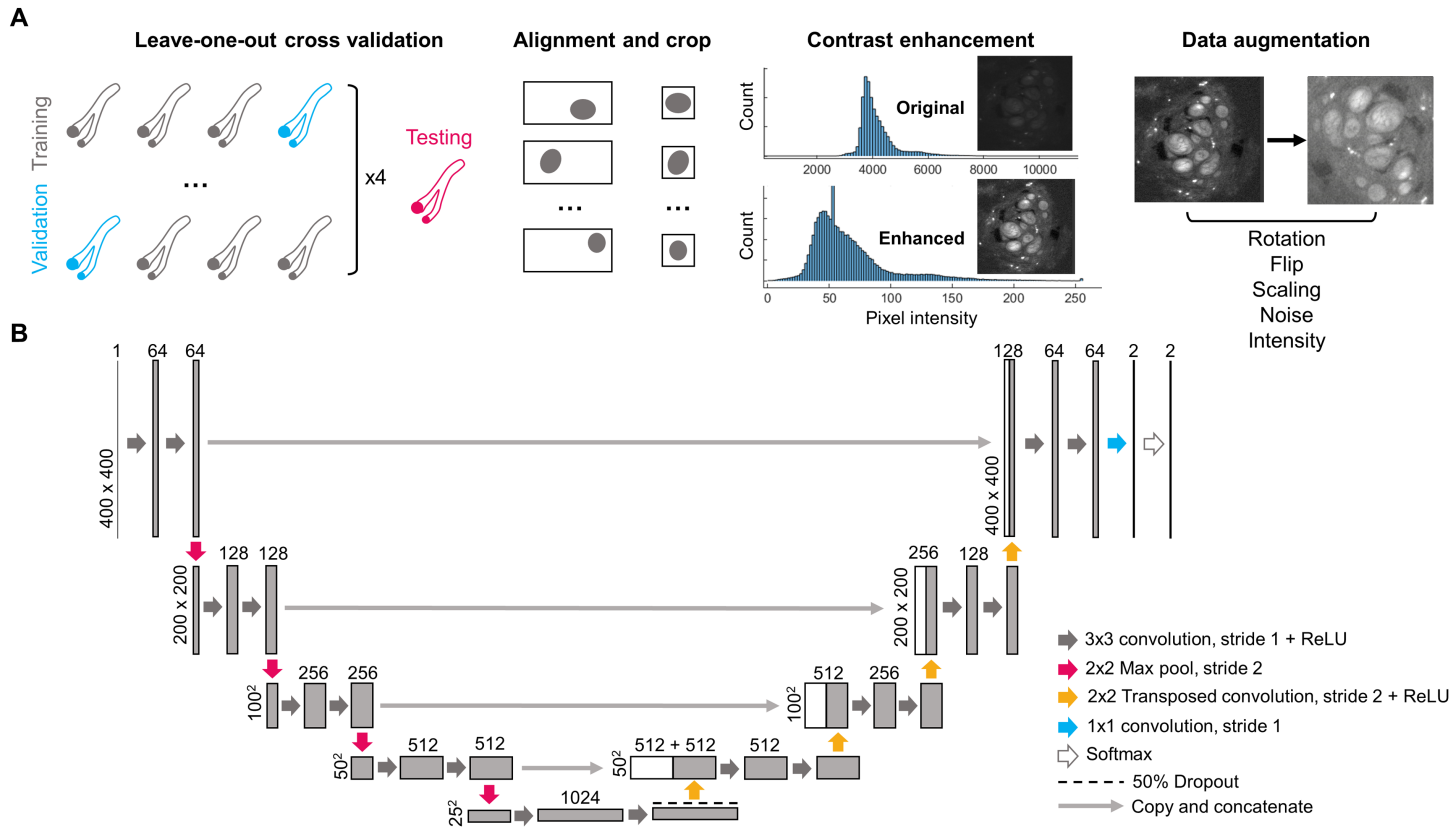
Pre-processing steps and architecture of deep learning network. **(A)** Four nerves (2 l, 2R, 3R, and 6R) were repeatedly separated as training and validation data using a leave-one-out cross-validation approach. A fifth nerve (6 l) was used for testing. As part of pre-processing, each image was centered on the epineurium and cropped, the contrast was enhanced, and the image was augmented via random rotations, flips, size changes, added noise and changes in pixel intensity. **(B)** Structure of the deep learning network, following the widely used U-Net architecture. Numbers above rectangles indicate the number of channels. Numbers on the left side indicate pixel dimensions. ReLU, Rectified linear unit.

A summary of the image pre-processing steps is shown in Figure 1A. Images were separated into training and validation sets based on a leave-one-out cross-validation approach (Sammut and Webb, 2010) where images obtained from three nerves were used at a time for network training, and images from one nerve were kept as validation. This was repeated four times so that the average performance from all four networks could be evaluated. Images from a separate fifth nerve were used for final testing. All images belonging to the same nerve were kept within the same training, validation or testing set since sequential images obtained in any given nerve are highly correlated with each other, and thus could lead to overfitting if separated across multiple sets.

Based on the manual segmentation of the nerve epineurium, the position of the epineurium centroid was calculated using a custom MATLAB script (MathWorks, Natick, MA, United States). Images were then automatically cropped to 400 × 400 pixels, centered around the nerve epineurium. Centering was applied to each microCT image slice and label pair separately as the position of the nerve in the imaging field-of-view changed from slice to slice.

In order to improve image uniformity, correct for staining heterogeneity between samples and increase contrast, pixel values in the microCT images were converted from 16-bit to 8-bit and image contrast was enhanced as follows: saturated pixels corresponding to staining artifacts (values >18,000, manually chosen threshold) were set to zero, after which the lowest 0.1% of pixels were set to 1, the highest 0.1% of pixels were set to 255, and the remaining pixels were rescaled linearly between 1 and 255. Before inputting into the CNN, images were converted to float 32 precision where the intensity of each image was normalized by dividing each pixel value by the mean intensity of the image.

Data augmentation was performed on-the-fly on each image during network training to increase the size of the training dataset and prevent network overfitting. Multiple compound transformations were randomly used on each image every epoch as follows: (1) a random rotation of angle 0°–270° (performed on 50% of images), (2) a vertical or horizontal flip (each 33% of images), (3) random scaling between 0.9 and 1.2x in size (66% of images), (4) random additive Gaussian noise with a mean of zero and a standard deviation randomly distributed between 0.001 and 0.003 (applied to all images), (5) random intensity variations added in the shape of an arbitrary function $f\left(x,y\right)=A*\sin\left(\mathrm{ax}\right)+B*\sin\left(\mathrm{by}\right)+1$ , where A, B, a, and b are random numbers in the range 0.001–0.3 (applied to 50% of images). All images were also smoothed with a Gaussian filter (standard deviation = 1 pixel) as a preprocessing step.

### 2.5. Network architecture and deep learning experiments

Network training was executed on the High Performance Computing cluster at Case Western Reserve University using a 48 GB A40 GPU (NVIDIA Corporation, Santa Clara, CA, United States). All deep learning networks were implemented and trained in MATLAB 2021a (MathWorks, Natick, MA, United States).

Convolutional neural networks with U-Net architecture (Figure 1B) were trained for fascicle segmentation. A Dice loss function was used to decrease the impact of class imbalance and improve segmentation results (in a typical 400 × 400 pixels image, only ~5% of pixels belong to the fascicle class). Networks were trained for 60 epochs. The initial learning rate was 5 × 10^−4^, and the learning rate was multiplied by 0.75 every 8 epochs. The mini-batch size was set to 20 images, and the Adam optimizer algorithm was used. An example training curve (for Network 1) can be see in Supplementary Figure S1.

### 2.6. Success metrics

The Dice similarity coefficient (DSC) was used to evaluate the network segmentation on the validation and testing sets on a per pixel basis:


(1) $$\mathrm{DSC}=\frac{2\left|\mathrm{True}\cap \mathrm{Predicted}\right|}{\left|\mathrm{True}\right|+\left|\mathrm{Predicted}\right|}$$

where True is the set of pixels identified as fascicles in the ground truth and Predicted is the set of pixels classified as fascicles by the deep learning network. While the Dice coefficient reflects the number of pixels correctly classified per image, it does not provide any information on the number of correctly identified fascicle cross-sections, nor on the occurrence of mistakes such as added, missed, merged or split fascicles. The accuracy of the network prediction was thus also evaluated on a per fascicle basis using an intersection-over-union (IoU) matrix (adapted from Caicedo et al., 2019, similar to Kirillov et al., 2019) where individual matrix elements are defined as:


(2) $$IoU_{i,j}=\frac{{\mathrm{True}}_{i}\cap {\mathrm{Predicted}}_{j}}{{\mathrm{True}}_{i}\cup {\mathrm{Predicted}}_{j}}$$

where IoU*_i,j_* is the intersection-over-union of a fascicle *i* (identified using connected components) in the ground truth image and a corresponding fascicle *j* (identified also using connected components) in the prediction image. True*_i_* is the set of pixels identified in the ground truth for fascicle *i*, while Predicted*_j_* is the set of pixels classified as fascicle *j* by the deep learning network. For analysis, a matrix IoU*_i x j_* can be calculated for all fascicle pairs *(i, j)*. Elements of IoU*_i x,j_* will be above a threshold *t* if a fascicle *i* in the ground truth has been correctly predicted as fascicle *j* by the network (true positive, TP). However, rows of IoU*_i x j_* with all elements equal to zero (or below threshold *t*) indicates missed fascicles in the prediction (false negatives, FN), and conversely columns of IoU*_i x j_* with all elements equal to zero (or below threshold *t*) indicates fascicles added in the prediction (false positives, FP). From these results, the fascicle F1-score reflects the number of correctly identified fascicles per image and is calculated as follow:

(3) $$F1_{\mathrm{fascicle}}=\frac{TP_{\mathrm{fascicle}}}{TP_{\mathrm{fascicle}}+\frac{1}{2}\left(FP_{\mathrm{fascicle}}+FN_{\mathrm{fascicle}}\right)}$$

Similarly, rows of IoU*_i x j_* with more than one non-zero element (>0.1) indicates a fascicle was mistakenly split by the network, and columns of IoU*_i x j_* with more than one non-zero element (>0.1) indicate that more than one fascicle were mistakenly merged by the network prediction. For this step, an effective IoU threshold was empirically derived to be 0.1 to include all cases of merges/split, not only when predicted and true fascicles have a significant overlap (IoU > 0.4). To further understand fascicles affected by the different types of mistakes, fascicles were also classified by sizes as follows: large (>300,000 μm^2^), medium (90,000–300,000 μm^2^), small (20,000–90,000 μm^2^), and tiny (<20,000 μm^2^).

### 2.7. Image processing software

Network training and network prediction was executed in MATLAB 2021a. Result visualizations were produced using ImageJ and MATLAB in 2D, and Amira (Thermo Fisher Scientific, Waltham, MA, United States) in 3D. Statistical plots were produced in MATLAB and Microsoft Excel (Microsoft Corporation, Redmond, WA, United States).

## 3. Results

### 3.1. U-Net successfully segments vagus nerve fascicles from microCT images

**Figure 2:**
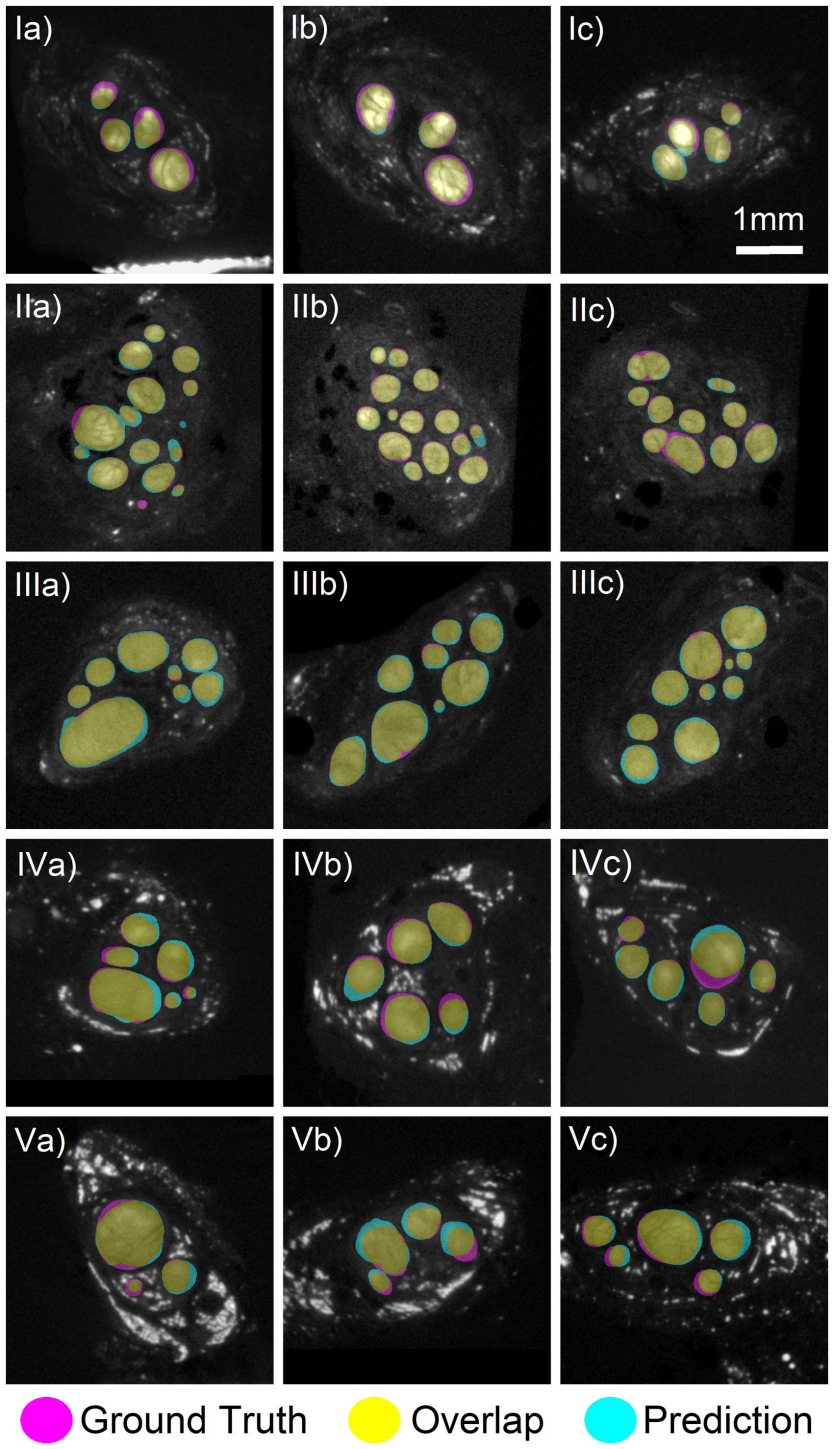
Comparison of deep learning segmentation to the ground truth segmentation. Networks 1–4 trained on three nerves with a fourth nerve kept out as validation. **(Row I)** Three slices from the validation specimen 2 L predicted using Net2. **(Row II)** Three slices from the validation nerve 2R using Net1. **(Row III)** Three slices from the validation nerve 3R using Net3. **(Row IV)** Three slices from the validation nerve 6R using Net4. **(Row V)** Three slices from the test nerve 6 L which was not included in any training of validation set. Prediction made by Net3. There was a high amount of overlap between the ground truth (magenta) and predicted (cyan) areas, colored in yellow. Images shown have an average Dice score of 0.88, 0.92, 0.92, 0.88, and 0.88, respectively, for each row.

Four deep learning networks (Net #1–4) were trained using leave-one-out cross-validation with the nerves 2R (501 images), 2 L (494 images), 3R (501 images), and 6R (501 images) kept out as validation in turn. As seen in Figure 2, the networks successfully segmented the nerve fascicles in all nerves, with high agreement between the predicted and ground truth fascicles. The network predictions were robust and unaffected by the wide variability across images: from small to large fascicles, from few to many fascicles, from high to low contrast images. The high variability in staining and imaging conditions across the nerves included in this study is shown in Supplementary Figure S2.

**Figure 3:**
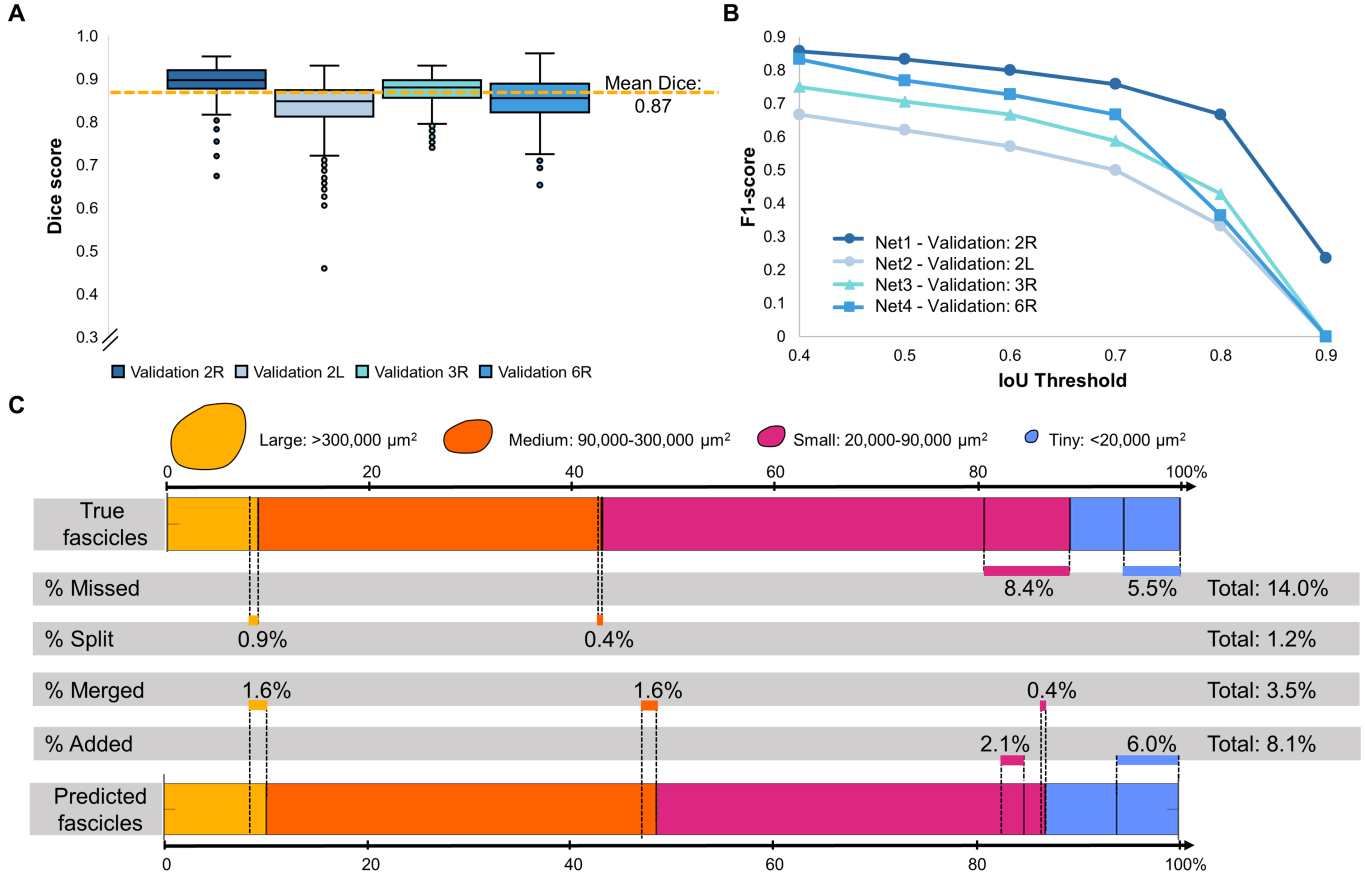
Evaluating segmentation accuracy on a per pixel and per fascicle basis. **(A)** Distribution of Dice coefficients for all four validation nerves, with 493–500 images per nerve. Mean Dice coefficient of 0.87 across all four nerves. **(B)** Per fascicle F1-score, indicating the prevalence of correctly (true positives) and incorrectly (false positives, false negatives) identified fascicles per image. A fascicle is identified as “true positive” if the predicted fascicle and the true fascicle overlap with an intersection-over-union (IoU) value above a certain threshold. **(C)** Size distribution of correctly and incorrectly identified fascicles for one example nerve (2R). Fascicles may have been missed (false negatives), incorrectly split, incorrectly merged, or incorrectly added (false positives) by the network prediction. Total percentage of fascicles affected by each error type is reported, with a breakdown into fascicle sizes affected by each error type (Large: yellow, Medium: orange, Small: fuchsia, Tiny: blue). Errors affecting <0.1% of fascicles are not reported. Data for other nerves (2 L, 3R, 6R) are reported in Supplementary Figure S4.

To quantify the performance of our networks, Dice coefficients were calculated between the predicted and ground truth fascicle masks (see Equation 1). The Dice coefficients of all images for all validation nerves can be seen in Figure 3A. The mean Dice coefficient was 0.87 across all four validation nerves (5th percentile: 0.77, 95th percentile: 0.93), which indicates high agreement between the network prediction and ground truth. Example of high, average and low Dice coefficient images are shown in Supplementary Figure S3, to demonstrate the range of segmentation performance exhibited at different numerical values.

However, Supplementary Figure S3 highlights that while the Dice coefficient is a measure of the overall fascicle area that was correctly segmented, it does not describe how individual fascicles were segmented from each other and it does not quantify the occurrences of errors such as missed or added fascicles, or accidental fascicle merges and splits. Examples of each type of fascicle segmentation error are shown in Supplementary Figure S5. Therefore, we also performed a per-fascicle analysis for all four validation nerves (similar to per-cell analysis of Caicedo et al., 2019). Using Equation (2), the IoU of each fascicle in the predicted and ground truth images was calculated, and fascicles in the predicted images were classified as true positives, false negatives, or false positives based on their level of overlap with the corresponding ground truth fascicle (i.e., if the IoU is above a threshold *t*). The F1_fascicle_ score was then calculated using Equation (3). The F1 scores obtained from each of the four validation nerves at increasingly high IoU threshold *t* can be seen in Figure 3B, and a visualization of different IoU per fascicle values and per-image F1 scores can be seen in Supplementary Figure S3. As an example, with a threshold of *t* = 0.4, fascicles overlapping with an IoU of *t* > 0.4 were classified as correctly detected by the network (true positive), while fascicles with an IoU *t* < 0.4 were classified as false positives or false negatives, and an average F1 score of 0.78 was obtained. However, with increasingly stringent IoU thresholds, fewer predicted fascicles were classified as true positives until true positive fascicles were only found in one nerve (2R) with *t* = 0.9. This result indicates that while the vast majorities of fascicles are correctly identified, the precise location of fascicle boundaries vary between the network prediction and the manual segmentation. Supplementary Figure S3 also highlights that smaller fascicles with fewer pixels are more sensitive to changes in individual IoU metrics, while larger fascicles with more pixels are less sensitive to IoU but have a larger impact on the per image Dice coefficient. Both metrics are thus essential to understand segmentation accuracy.

To quantify the type of mistakes made by the network when identifying individual fascicles, we broke down the type of errors (fascicles falsely added, missed, merged, or split) as a function of fascicle size (see Figure 3C for nerve 2R, see Supplementary Figure S4 for all other nerves). In nerve 2R, a small minority (0.4–1.6%) of large (>300,000 μm^2^) and medium fascicles (90,000–300,000 μm^2^) were accidentally split or merged, and <0.1% were mistakenly added or missed by the algorithm. On the other hand, fascicles incorrectly added or missed by the algorithm were more common in the small (20,000–90,000 μm^2^) and tiny fascicles (<20,000 μm^2^), where a total of 14% of fascicles were missed, and 8.1% were incorrectly added. A few of the small fascicles (0.4%) were also incorrectly merged. As seen in Supplementary Figure S4, this distribution of added/missed/split/merged fascicles varied between nerves. For example, validation nerve 3R had virtually no mistakenly split fascicles (<0.1%) across all sizes, while a significant percentage of large fascicles (6–8%) were incorrectly merged by the algorithm in validation nerve 2 l, 3R, and 6R.

### 3.2. U-Net segmentation generalizes well to other nerves not included in the training or validation datasets

**Figure 4:**
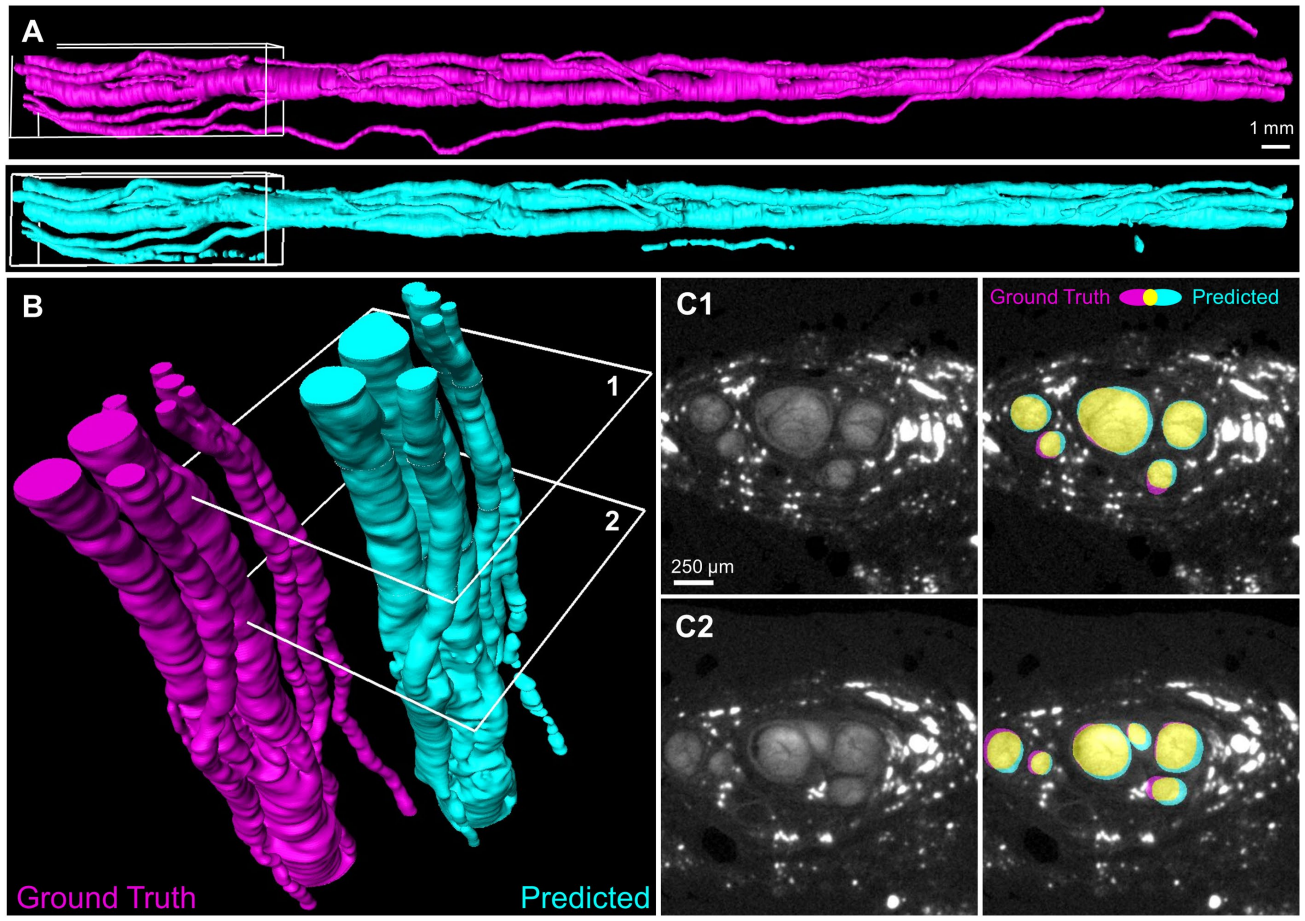
Deep learning segmentation of the full three-dimensional fascicles of a human vagus nerve. **(A)** Volume surfaces recreated from sequential 2D automated segmentation of a 4.4 cm nerve segment by Network 3. Magenta: manually generated ground truth of the testing nerve 6 L. Cyan: network prediction. **(B)** Region of interest (white boxes in **A**) showing individual nerve fascicles. **(C)** Two example slices (identified as 1 and 2 in **B**) with true and predicted segmentation shown in magenta and cyan. Yellow indicates overlap. Dice coefficients = 0.90 and 0.85 for slice 1 and 2, respectively.

We used our network to predict fascicle segmentation on a separate test nerve that was not included in the training or validation set. The prediction was made on a 4.4 cm vagus nerve segment (6 L) comprised of 444 2D slices using Network 3 (Figure 4). The predicted fascicle segmentation strongly resembled the ground truth segmentation of the same nerve, apart from one small diameter fascicle (Figure 4A) which appeared to track alongside the nerve and was only detected by the network at a few discrete intervals. As seen in the close-up rendering of one end of the nerve (Figure 4B), the individual nerve fascicles were correctly detected. Importantly, the frequent fascicle merging and splitting previously characterized in the vagus nerve (Upadhye et al., 2022) were visible along the nerve length. Two example slices (Figure 4C) demonstrate the high level of overlap between the predicted and ground truth images, with individual Dice coefficients of 0.90 and 0.85. No additional post-processing was performed on the network segmentation, but additional refinements could be made manually for inputs to computational modeling (Marshall et al., 2022).

**Figure 5:**
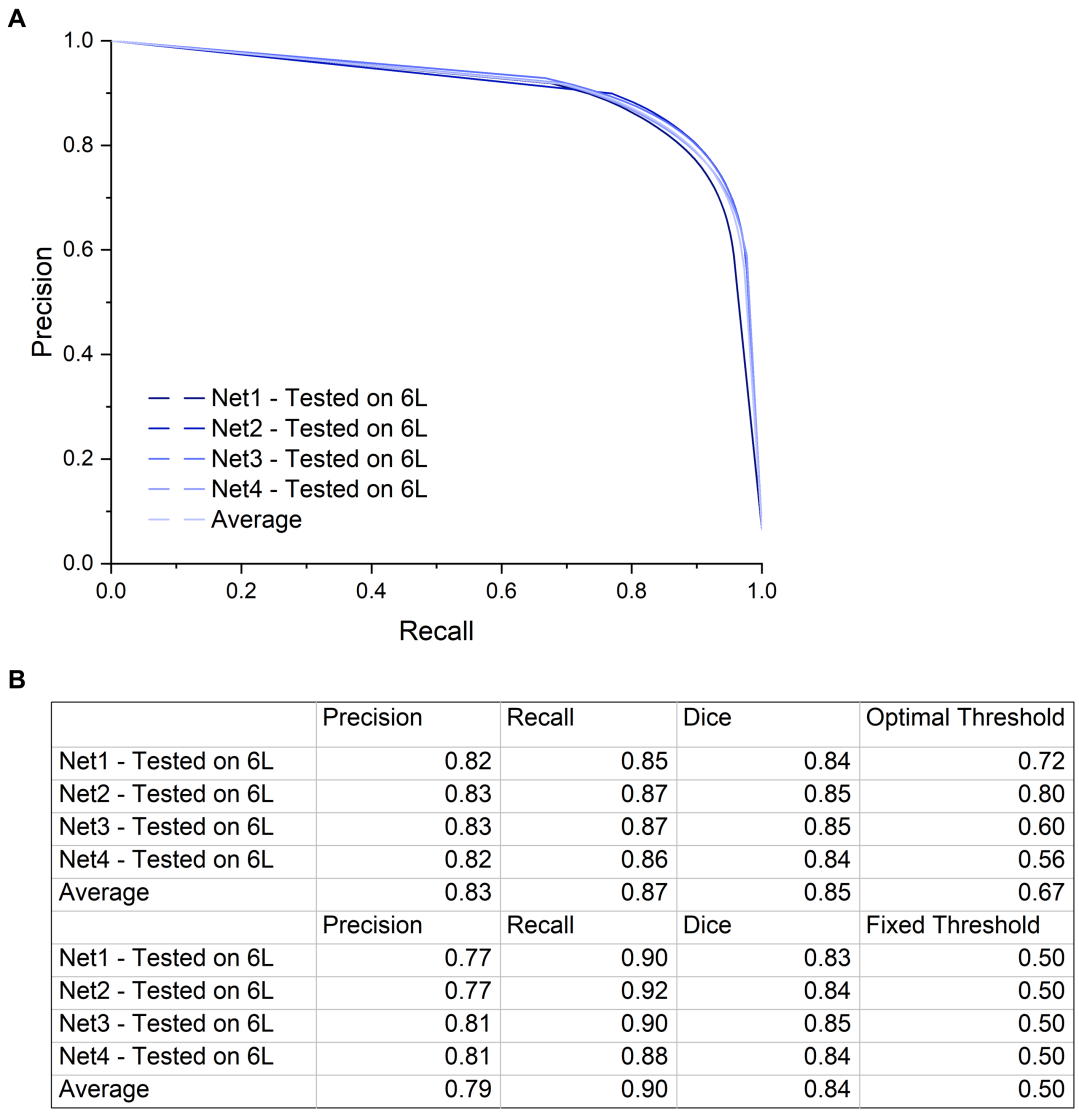
Precision-Recall curve and a table with the precision and recall values at the highest Dice coefficient. **(A)** Precision-Recall curve with a large AUC (Area Under Curve) indicating low numbers of false positives and false negatives. Although training specimens are different from one another and from the test specimen in terms of anatomy and contrast, performance of the networks on the test dataset is similar with high accuracy. The precision-recall curve was generated by applying a variable threshold from 0 to 1 with a step size of 0.02 to the output of each of the four networks and calculating the precision and recall at every step. **(B)** The first four rows of this table shows the precision and recall values using the optimal threshold value at which the highest Dice coefficient is calculated for each network. The next four rows show the precision, recall and Dice coefficient at a fixed threshold of 0.5. There is not a big difference in network performance at the optimal thresholds compared to the fixed threshold.

To quantify the ability of our network to segment the microCT images of a nerve that were not used in the training or validation of the CNN, all four trained networks were individually tested on nerve 6 L, and the per-pixel recall, precision, and Dice coefficient were calculated on the resulting images (Figure 5). All four networks performed similarly when tested on nerve 6 l, with an average Dice coefficient of 0.85. This demonstrates that even with the high variability in staining intensity, nerve morphology, and nerve appearance in our limited dataset, the composition of the training dataset has little influence on the end performance for segmenting a new nerve. This was further supported by the shape of the precision-recall curve, showing an AUC (area under curve) value close to 1 (Figure 5A) for each instance and the average.

### 3.3. Network segmentation vastly improves throughput

Automated fascicle segmentation is essential for volumetric microCT images, as manual segmentation is highly labor- and time-intensive. From our experience using a typical segmentation software, it took approximately 40 h for a trained user to manually segment all the fascicles in 501 image slices. This is a significant time commitment which slows down the data pipeline from image acquisition to fascicle analysis and modeling. Considering that multiple specimens with 501 slices (5 cm, covering the surgical window) are required for computational modeling, the manual segmentation process is months-long. In addition, in order to further characterize of the vagus nerve morphology throughout the body, the desired segmentation nerve length could be much longer.

While computing speeds can vary from computer to computer, our deep learning network trained in approximately 173 min (using 1,500 images from three nerves for 60 epochs and a mini-batch of size 20), and it can segment the fascicles in one image in 0.05 s. Overall, a 5 cm nerve segment can be segmented in an average of 24.3 s. This is four orders of magnitude faster than manual segmentation. Even considering that a user might want to apply manual correction after automated segmentation, the time saved is considerable and enables fast transition times between nerve imaging and nerve modeling.

## 4. Discussion

Here, we developed a U-Net convolutional neural network to automate segmentation of fascicles in microCT of human vagus nerve. Once trained, the network was capable of segmenting fascicles nearly 4 orders of magnitude faster than manual segmentation. The resulting segmentations were assessed for accuracy in multiple ways, including the standard performance metric, the Dice similarity coefficient, as well as a newly developed metric to assess fascicle detection, the combination of per fascicle Intersection over Union (IoU) and per image F1 score. While the standard metrics suggest generally good accuracy (Dice coefficient 0.87), the fascicle metrics suggest that the segmentations tend to under-detect “small” or “tiny” fascicles, in some cases, >10%. Even so, performance is generally good and provides a rapid first approximation that is similar in appearance to the ground truth segmentations (e.g., Figure 4A). While we did not specifically measure it here, the time savings from automatic segmentation is so great that a user could likely apply manual revisions/corrections and still provide net benefits over purely manual segmentation.

### 4.1. First demonstration of U-Net-based segmentation of peripheral nerve fascicles from microCT images

This is the first demonstration of automated vagus nerve fascicle segmentation from microCT images using U-Net. While there has been ongoing interest in segmenting peripheral nerves imaged with ultrasound (Wang et al., 2019; Horng et al., 2020; Kim et al., 2022) and magnetic resonance imaging (Balsiger et al., 2018), the focus of these studies has been on identifying and locating the peripheral nerves within the surrounding tissues to aid clinicians with the diagnosis of neurological problems. As a result, the segmentation algorithms used in these studies focused on segmenting the nerve from the background structures. In comparison, recent efforts to image pig and human vagus nerves used excised nerves and higher resolution approaches (e.g., microCT) (Thompson et al., 2020; Upadhye et al., 2022; Jayaprakash et al., 2023). Here, we aimed to automatically segment individual nerve fascicles and to characterize segmentation accuracy on a per-pixel and per-fascicle basis, with an ultimate goal of accelerating the study of human vagus nerve morphology to improve VNS therapies.

We chose the U-Net architecture as a benchmark for automated fascicle segmentation since the encoder-decoder architecture of U-Net and other U-Net variations (such as nnU-Net) are easy to implement and have been highly successful in a wide array of medical image segmentation (Isensee et al., 2021). U-Net also has a straightforward expansion to three-dimensional volumes, 3D U-Net (Çiçek et al., 2016), which could provide an easy transition toward volumetric segmentation of fascicles in the future. It is expected that any attempts toward improved CNN architecture for fascicle segmentation will have to outperform U-Net accuracy, thus the current demonstration of U-net acting as a benchmark.

### 4.2. U-Net-based deep learning approach will improve computational modeling of VNS

Computational modeling of neuromodulation therapies provides an important suite of tools to analyze neural responses to stimulation and to design improved approaches (Musselman et al., 2021). However, the accuracy of outputs from all models relies on the accuracy of the inputs. Computational models of peripheral nerve stimulation—across different nerves and species—typically define the nerve morphology using a cross section of the nerve and fascicle boundaries that is extruded longitudinally (e.g., Helmers et al., 2012; Pelot et al., 2017; Bucksot et al., 2019). The cross section may be defined from segmented histology (Pelot et al., 2020) or by using a simplified representation. Thus, the models assume that changes in fascicle morphology along the length affected by neural stimulation are negligible.

The assumption of constant cross-sectional morphology along the nerve length may be appropriate for certain nerves, such as the sciatic or femoral nerve (Gustafson et al., 2009, 2012). However, microCT of human vagus nerves recently showed that the fascicles split and merge every ~560 μm, and that there is substantial variability in fascicle size, number, and location within and across samples (Upadhye et al., 2022). Due to the heterogeneous electrical properties of different neural tissues, these morphological parameters affect the electric field, and thus affect the resulting neural responses (Pelot et al., 2019; Davis et al., 2023). Further, the tortuous fiber paths and smaller electrode-fiber distances at certain locations along the nerve would result in lower activation thresholds (Marshall et al., 2022). Therefore, assuming a constant cross section for human vagus nerves may be inaccurate for modeling the response to VNS.

More realistic models of the vagus nerve that take into account variations in fascicle morphology are thus likely required to achieve precise prediction of VNS performance. These models will serve to simulate population responses (e.g., Musselman et al., 2023), study mechanisms of action (e.g., Davis et al., 2023), and design improved electrode geometries, electrode placements, and stimulation parameters (e.g., Schiefer et al., 2008; Wongsarnpigoon and Grill, 2010; Aristovich et al., 2021). There is thus substantial interest in characterizing the fascicular morphology of the vagus nerve along its length and in a larger number of human cadaver subjects (NIH, 2022). However, to accomplish these goals, fast and accurate fascicle segmentation are necessary to expand our understanding of the functional organization of the vagus nerve (Ravagli et al., 2021; Thompson et al., 2022; Upadhye et al., 2022; Jayaprakash et al., 2023).

High resolution imaging methods like microCT provides detailed information about fascicular morphology along the length of the vagus nerve (Thompson et al., 2020; Upadhye et al., 2022; Jayaprakash et al., 2023). Thompson et al., imaged peripheral nerves (rat sciatic and pig vagus) using microCT and were able to manually segment and trace fascicles in their samples (Thompson et al., 2020). Jayaprakash et al. (2023) imaged the pig vagus nerve for the purpose of tracing “organotypic” fascicular connectivity. By taking into account the fascicle morphology over the length of the nerve, microCT imaging has the potential to improve computational models of VNS.

In addition to the impact on computational modeling there is substantial merit and potential impact to further elucidating the micro-anatomy of the vagus nerve. The parasympathetic autonomic system is very complex and involved in many functions within the body, and may therefore underpin multiple diseases (Andersson and Tracey, 2012; De Couck et al., 2012; Breit et al., 2018).

### 4.3. Limitations and future directions

Our approach may be improved by training a network to segment the epineurium in addition to fascicles. Epineurium segmentation provides morphological quantification and inputs to computational modeling on the size of the nerve, which is important for designing cuff electrodes that are appropriately sized and for predicting activation thresholds with correct electrode-fiber distances. However, additional staining procedures are needed to enhance the contrast of the epineurium.

It is important to note that the segmentations are performed on dissected and processed tissue which inevitably causes volume shrinkage. Therefore, our segmentations underestimate fascicle dimensions and it would be necessary to compensate for shrinkage if utilizing this data for subsesquent electrode design efforts. Past publications have estimated that tissue shrinkage is on the order of 15–30% (Pelot et al., 2020).

Fascicle sizes and locations in adjacent cross sections of the nerve are also highly spatially correlated. Although our network performed well on both the validation and test nerves, its performance could improve if a 3D network approach were taken (Çiçek et al., 2016). We sampled nerves from de-identified cadavers, but in future studies, demographic information may be leveraged to study potential effects on nerve morphology.

Staining with osmium tetroxide enhances the brightness of the fascicles in images, primarily through its reaction with lipid-containing myelin (Kiernan, 2007), but the osmium was not uniformly absorbed in all specimens and along the length of the nerve in single specimens, resulting in areas with non-uniform brightness. This limitation may be able to be overcome with additional optimization of the staining procedures and control of the time from death to embalming, dissection, and staining. Nonetheless, despite variability in tissue contrast, our deep-learning approach seemed to be robust and made similarly accurate predictions regardless of which nerves were included or excluded of the training dataset.

Even though our ground truth segmentations were performed by trained ‘experts’, there is an inherent risk that some of the smaller fascicles could have been missed. We believe this is particularly possible as some of the smaller fascicles would be on the order of 3 × 3 pixels (or 30 × 30 μm). Therefore, it is possible that improvements in the ground truth segmentation, aided by higher resolution imaging, may yield better performance of the network detection of smaller fascicles. In general higher quality images improve automatic detection method performance (Sabottke and Spieler, 2020).

Lastly, lipid deposits and/or precipitates of osmium tetroxide generate high pixel intensity artifacts. While these were occasionally misinterpreted by the network as fascicles, these are easy to identify and remove during manual clean-up processing.

## 5. Conclusion

Understanding the fascicular organization of the vagus nerve is an important step in the development and optimization of neuromodulation devices that selectively activate the nerve’s diverse functional pathways.

We demonstrated that a U-Net CNN successfully segments fascicles from microCT images of the human vagus nerve. Further, we demonstrated that U-Net segmentation generalizes well to other nerves not included in the training or validation datasets. This automated segmentation approach vastly improved throughput compared to manual segmentation.

While the network’s Dice coefficient (0.87) suggests moderate-to-high performance, the segmentations are not perfect. To supplement typical segmentation metrics, we developed a fascicle-wise detection metric that categorized various types of errors (falsely missed, added, split or merged fascicles). This network and the calculated performance metrics herein set a first benchmark, using a standard U-Net CNN, for the application of deep-learning algorithms to segment fascicles from microCT images. The process may be further optimized by refining tissue staining methods, modifying network architecture, and expanding the ground-truth training data sets. The improvement in processing time has significant implications for the consortium of researchers currently engaged in mapping the entire length of the vagus nerve, as manually segmenting long nerves would be otherwise untenable.

## Data availability statement

The datasets presented in this study can be found in online repositories. The names of the repository/repositories and accession number(s) can be found at: http://www.sparc.science.10.26275/cise-ea9p.

## Author contributions

OB and ML-L were principally responsible for implementing and performing deep learning experiments, result analysis, figure conceptualization, and writing of the manuscript. CK and AU collected the primary datasets. DM and NP contributed to the project conception, data review, funding acquisition, careful review and manual editing of the ground-truth image dataset, and writing of sections of the paper. KL and KG contributed to the review and interpretation of the data, writing and editing of the final manuscript. DW, MJ, and AS were involved in all aspects of the data collection, funding acquisition, writing, reviewing, and editing of the manuscript. All authors contributed to the article and approved the submitted version.

## Funding

This work was supported by NIH’s Stimulating Peripheral Activity to Relieve Conditions programs 75N98022C00018 and OT2 OD025340, US Dept. of Veterans Affairs 1IS1BX004384, the Cleveland VA APT Center, and Case Western Reserve University. The opinions expressed in this article are the author’s own and do not reflect the view of the National Institutes of Health, the Department of Health and Human Services, or the United States government. Maryse Lapierre-Landry is supported by the American Heart Association Postdoctoral Fellowship Grant #916963, 2022–2023.

## Conflict of interest

The authors declare that the research was conducted in the absence of any commercial or financial relationships that could be construed as a potential conflict of interest.

## Publisher’s note

All claims expressed in this article are solely those of the authors and do not necessarily represent those of their affiliated organizations, or those of the publisher, the editors and the reviewers. Any product that may be evaluated in this article, or claim that may be made by its manufacturer, is not guaranteed or endorsed by the publisher.
