# Supplementary material for: Deep-learning segmentation of fascicles from microCT of the human vagus nerve
Source: Front Neurosci. 2023 May 10;17:1169187. doi: 10.3389/fnins.2023.1169187 (PMC10275336; doi:10.3389/fnins.2023.1169187)
Supplement: Supplementary file 1 [file Data_Sheet_1.pdf]

Supplementary Figures

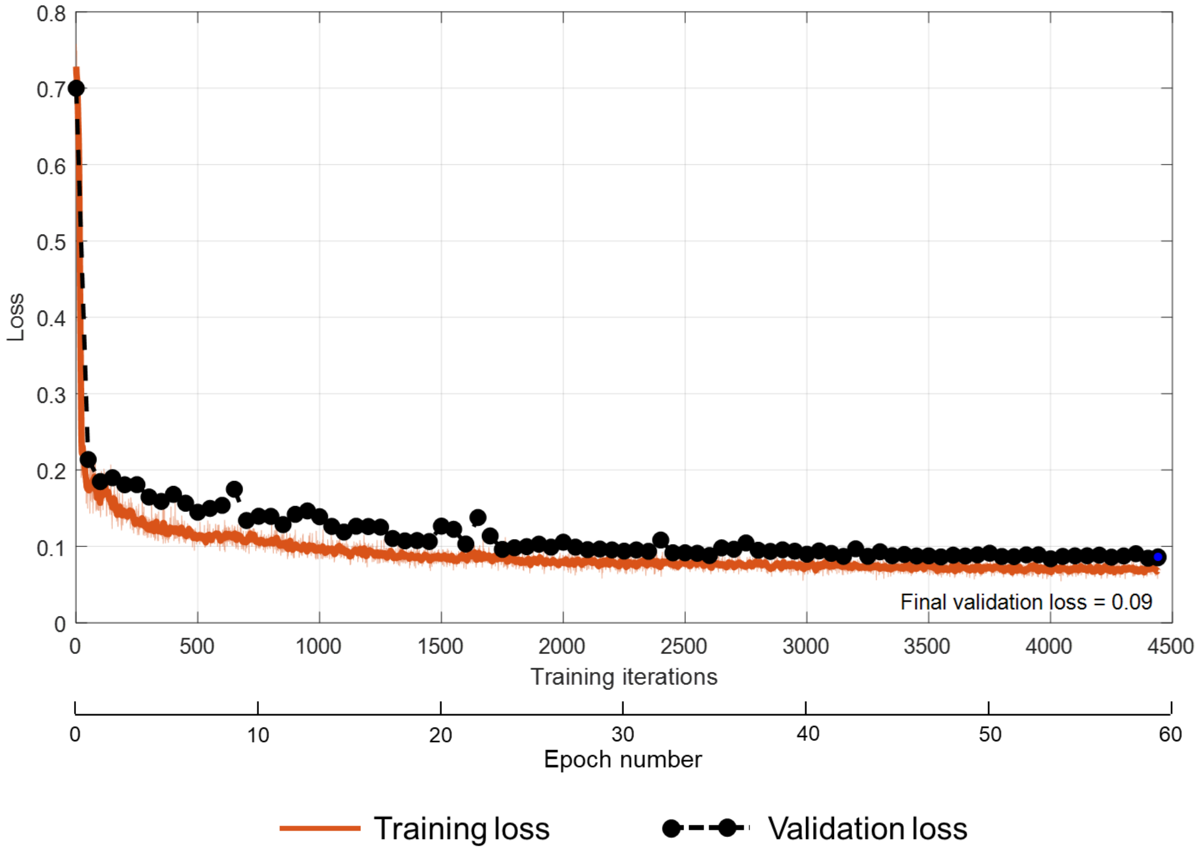

Supplementary Figure 1: Training and validation loss curves. Example loss curves for Network 1 trained on nerve 2L, 3R and 6R, and validated on nerve 2R using a Dice loss function. The network was trained on 60 epochs after which the lack of improvement in the validation loss led the end of network training. The final validation loss is 0.09.

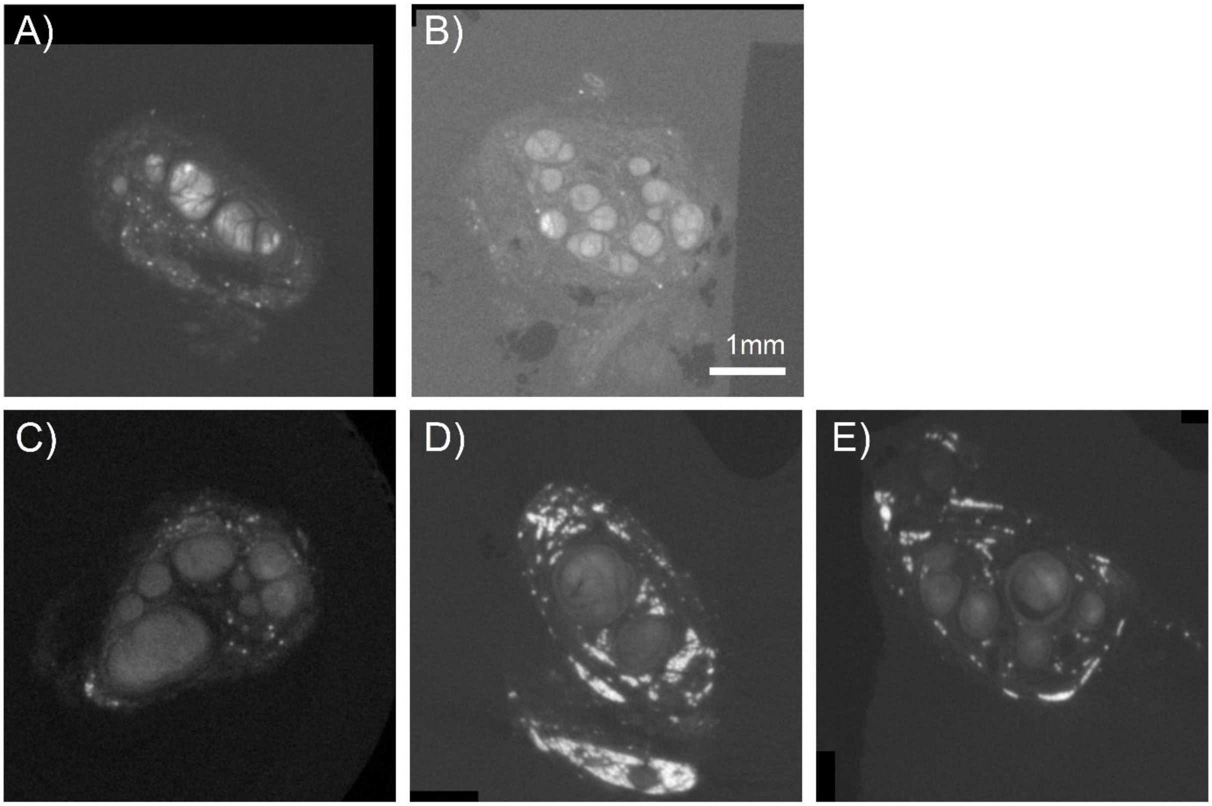

Supplementary Figure 2: Example cross sections of five human cervical vagus nerves showing the range of variability between sample contrast, fascicle count and diameters. A) 2L, **B)** 2R, **C)** 3R, **D)** 6R, **E)** 6L. Nerves A to D were used for training four networks with leave-one-out cross-validation. The specimen that image E came from was used for testing. For visualization purposes, the brightness/contrast for each of the displayed images was 'auto-adjusted' using ImageJ's default settings.

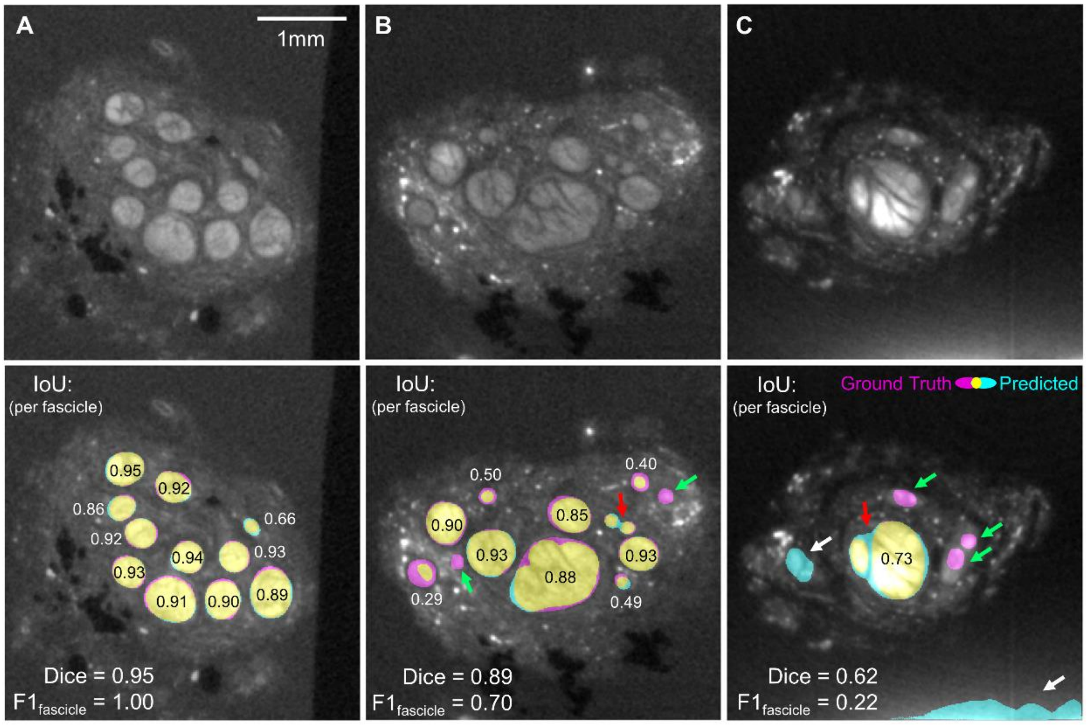

Supplementary Figure 3: Illustration of the different success metrics: Dice coefficient, per-fascicle intersection-over-union (IoU), and fascicle F1-score. Example of nerve segmentation from **A)** best (99.9th percentile of Dice coefficient), to **B)** typical (65th percentile Dice coefficient, below average F1 score), to **C)** worst (0.1st percentile of Dice coefficient). Dice coefficients are calculated for the whole image, IoUs are calculated for each fascicle, and F1-scores are calculated per image for a threshold of IoU > 0.4 to determine true positive fascicles. No IoU values are displayed for merged, missed or added fascicles. Magenta: ground truth. Cyan: network prediction. Yellow: Overlap. Red arrows: merged fascicles. Green arrows: missed fascicles. White arrows: added fascicles.

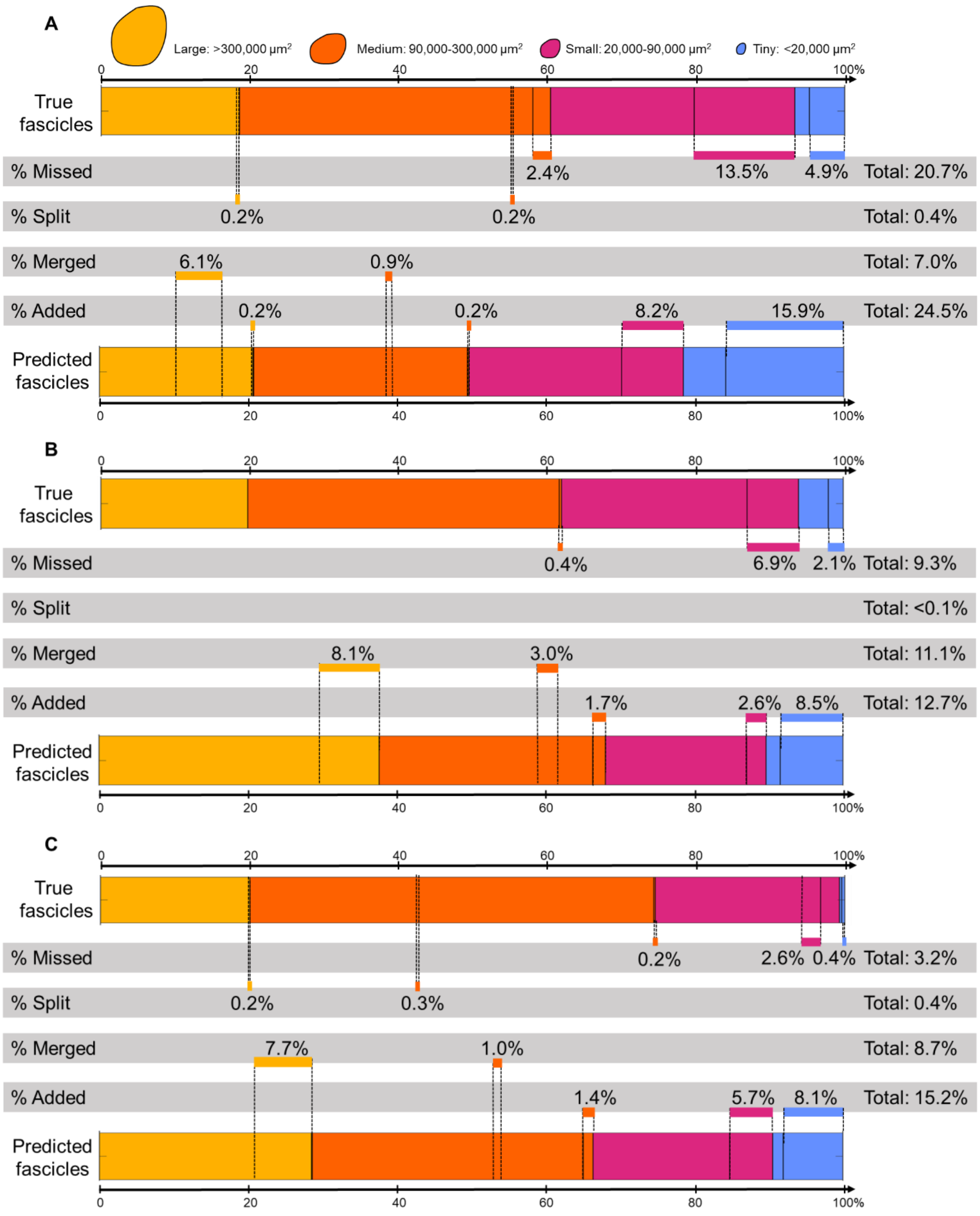

Supplementary Figure 4: Size distribution of correctly and incorrectly identified fascicles in three nerves from the validation set. A) Nerve 2L, **B)** Nerve 3R, **C)** Nerve 6R. Fascicles may have been missed (false negatives), incorrectly split, incorrectly merged, or incorrectly added by the network prediction (false positives). Total percentage of fascicles affected by each error type is reported, with a breakdown into fascicle sizes affected by each error type (Large: yellow, Medium: orange, Small: fuchsia, Tiny: blue). Errors affecting <0.1% of fascicles are not reported.

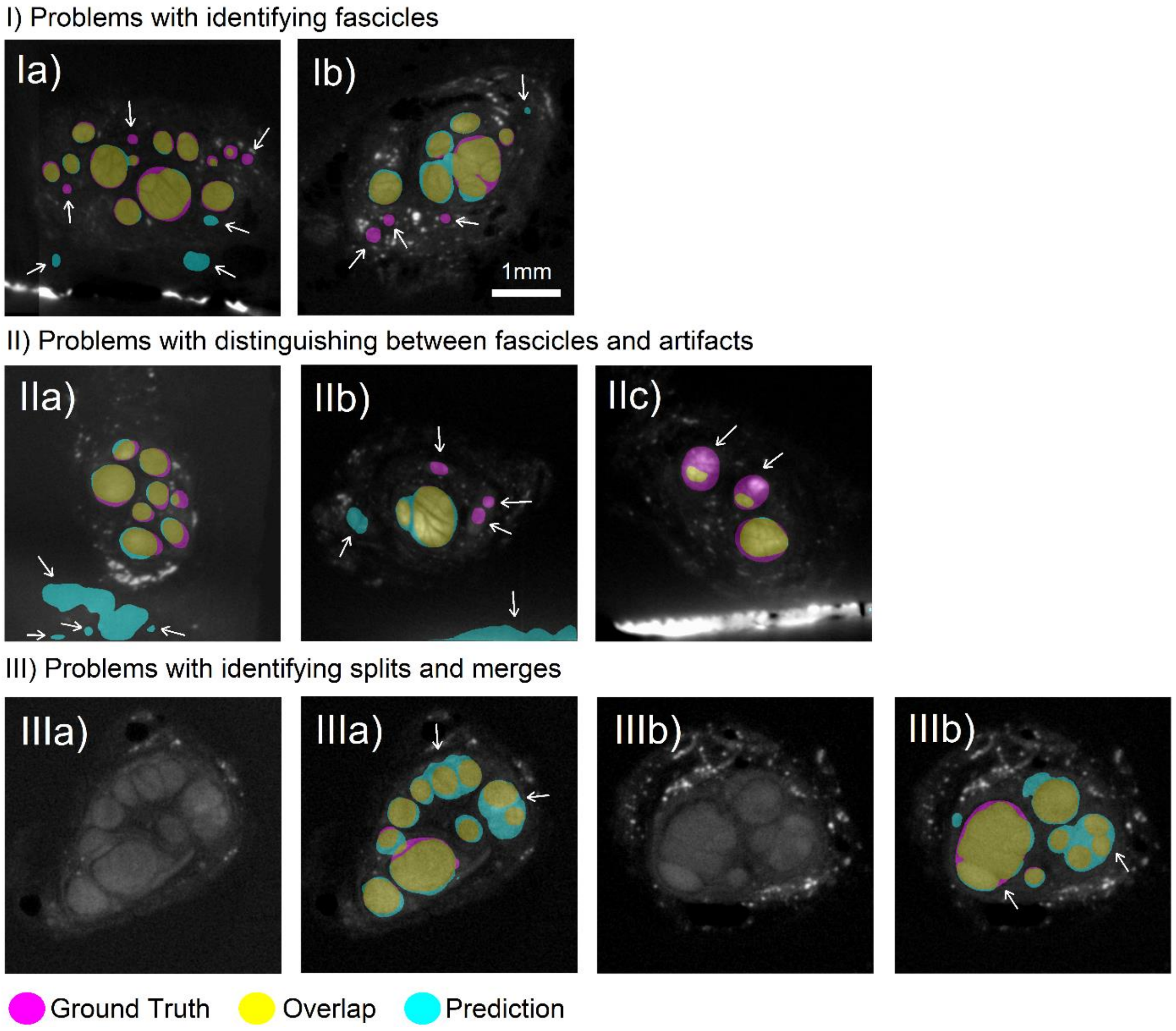

Supplementary Figure 5: The three most common segmentation errors. Row I) Two slices where small (20,000 – 90,000 μm^2) and tiny (< 20,000 μm^2) fascicles were either missed (pink "ground truth" fascicle without yellow overlap) or added (cyan "prediction" fascicle without yellow overlap). The average Dice coefficient for this row is 0.86. **Row II)** Three slices with bright line artifacts. This artifact is caused by the $BaSO_4$ (barium sulfate) applied onto the paraffin mold as a distance marker to assist in scanning long specimens. In these images, this artifact is either identified as a fascicle (IIa and IIb, cyan areas at the bottom left and right), or fascicles that are as bright as this artifact are ignored (IIc, fascicles in magenta). The average Dice coefficient for this row is 0.64. **Row III)** Splits and merges in fascicles are continuous events along the longitudinal of the nerve. Due to low contrast from staining and down-sampling of large number of images, there are differences between the trained user's and the network's decision on when a split or merge occurs. In both examples, clusters of fascicles were segmented as a single fascicle (cyan) by the user and separate fascicles (yellow overlap) by the network. The average Dice coefficient for this row is 0.84.
